# Imidazole catalyzes chlorination by unreactive primary chloramines

**DOI:** 10.1016/j.freeradbiomed.2015.01.026

**Published:** 2015-02-04

**Authors:** Margo D. Roemeling, Jared Williams, Joseph S. Beckman, James K. Hurst

**Affiliations:** a Department of Biochemistry and Biophysics, Oregon State University, Corvallis OR, USA; b Environmental Health Sciences Center, Oregon State University, Corvallis OR, USA; c Linus Pauling Institute, Oregon State University, Corvallis OR, USA

**Keywords:** Chloramines, fluorescein, imidazole catalysis, oxidative stress, phenolic chlorination, transchlorination dynamics and equilibria

## Abstract

Hypochlorous acid and simple chloramines (RNHCl) are stable biologically-derived chlorinating agents. In general, the chlorination potential of HOCl is much greater than that of RNHCl, allowing it to oxidize or chlorinate a much wider variety of reaction partners. However, in this study we demonstrate by kinetic analysis that the reactivity of RNHCl can be dramatically promoted by imidazole and histidyl model compounds via intermediary formation of the corresponding imidazole chloramines. Two biologically relevant reactions were investigated—loss of imidazole-catalyzed chlorinating capacity and phenolic ring chlorination using fluorescein and the tyrosine analog, 4-hydroxyphenylacetic acid (HPA). HOCl reacted stoichiometrically with imidazole, N-acetylhistidine (NAH), or imidazoleacetic acid to generate the corresponding imidazole chloramines which subsequently decomposed. Chloramine (NH_2_Cl) also underwent a markedly accelerated loss in chlorinating capacity when NAH was present, although in this case NAHCl did not accumulate, indicating that the catalytic intermediate must be highly reactive. Mixing HOCl with 1-methylimidazole (MeIm) led to very rapid loss in chlorinating capacity via formation of a highly reactive chlorinium ion (MeImCl^+^) intermediate; this behavior suggests that the reactive forms of the analogous imidazole chloramines are their conjugate acids, e.g., the imidazolechlorinium ion (HImCl^+^). HOCl-generated imidazole chloramine (ImCl) reacted rapidly with fluorescein in a specific acid-catalyzed second order reaction to give 3′-monochloro and 3′,5′-dichloro products. Equilibrium constants for the transchlorination reactions: HOCl + HIm = H_2_O + ImCl and NH_2_Cl + HIm = NH_3_ + ImCl were estimated from the dependence of the rate constants upon [HIm]/[HOCl] and literature data. Acid catalysis again suggests that the actual chlorinating agent is HImCl^+^; consistent with this interpretation, MeIm markedly catalyzed fluorescein chlorination by HOCl. Time-dependent imidazole-catalyzed HPA chlorination by NH_2_Cl was also demonstrated by product analyses. Quantitative assessment of the data suggests that physiological levels of histidyl groups will react with primary chloramines to generate a flux of imidazole chloramine sufficient to catalyze biological chlorination via HImCl^+^, particularly in environments that generate high concentrations of HOCl such as the neutrophil phagosome.

## INTRODUCTION

It is generally accepted that neutrophils activated in aerobic environments generate microbicidal levels of HOCl through the concerted action of a phagosomal NADPH oxidase (NOX-2) and myeloperoxidase (MPO) [1–3]. Hypochlorous acid is sufficiently reactive toward biological targets [4,5] that it is not thought to persist in the phagosomal environment much beyond cessation of the NOX-2 respiratory burst (at about 20 min post-activation) [6,7]. However, chloramines formed from endogenous amines are among the immediate reaction products [5,8–11]; these can be equally bactericidal [9,10], but are considerably less reactive than HOCl [11] and *de facto* more selective in their reactions with potential reductants. As a consequence, chloramines can accumulate and extend the duration of chlorinating capacity of activated neutrophils. Evidence that a long-lasting pool of chlorinating agents is generated within the neutrophil phagosome following the respiratory burst includes chlorination of tyrosyl rings to form stable 3-monochloro- and 3,5-dichloro products [12–14] and bleaching of green fluorescent protein (GFP) expressed within the cytosol of phagocytosed bacteria [15,16]; both of these reactions appear to be specific for HOCl, but both are observed to occur as late as 1–2h post-activation of the neutrophil.

These reactions pose something of a puzzle since, under physiological conditions, tyrosine and GFP are moderately reactive toward HOCl, but virtually unreactive toward simple chloramines, i.e., NH_2_Cl or RNHCl (where R is an alkyl or aminoacyl substituent). Furthermore, the amount of HOCl present in equilibrium with endogenous chloramines under physiological conditions is expected to be in the low nanomolar range, which is far too low to account for observed rates of tyrosine formation and GFP bleaching.^1^ This conclusion is confirmed by the experimental design of the extracellular studies themselves, in which the chloramines are typically formed by reaction of HOCl with the amines, in essence achieving the equilibrium distribution of HOCl and chloramine before introduction of the HOCl-sensitive reactant. The absence of detectable reaction under these conditions therefore precludes the possibility that reaction in cellular environments arises from residual HOCl.

**Fig. 1:**
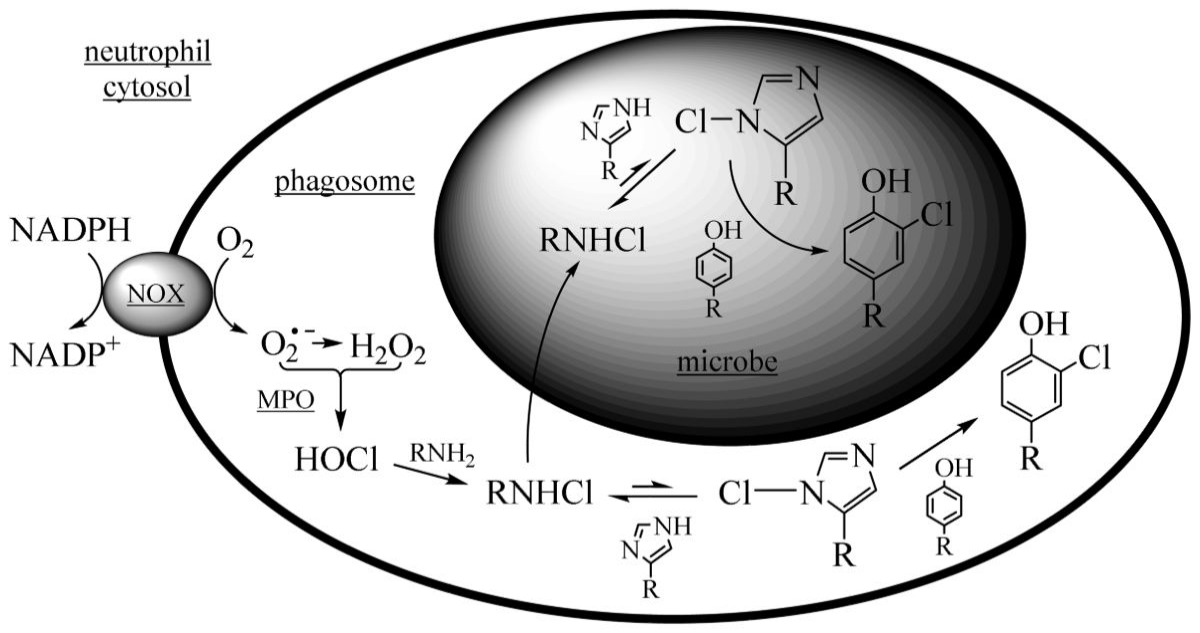
Hypothetical scheme for intraphagosomal catalysis of chlorination reactions by imidazole chloramine. Hypochlorous acid generated by the combined action of NADPH oxidase (NOX) and myeloperoxidase (MPO) reacts with primary amines to form chloramines. Following depletion of reactive nucleophiles by both of these chlorinating agents (reactions not shown), a chloramine pool is established. Transchlorination equilibration with imidazole-containing compounds leads to formation of small amounts of imidazole chloramines, which are sufficiently reactive within the biological milieu to chlorinate weaker nucleophiles such as phenolic compounds.

If not HOCl or simple chloramines, how then does one account for the relatively slow, continued chlorinating capacity demonstrated within the neutrophil phagosome? Work from the Davies group has shown that imidazole chloramines formed by reaction of imidazole-containing compounds with HOCl retain a chlorinating capacity that is much greater than simple chloramines [4,19,20]; for these compounds, relative chlorination rate constants approach within one or two orders of magnitude that of HOCl itself. One attractive possibility therefore is that endogenous imidazole groups may catalyze transchlorination reactions from the chloramine pool to receptive molecules or functional groups within the phagosomal microenvironment (e.g., as envisioned in Fig. 1).

In the present study, we investigate further the nature of imidazole catalysis of several reactions involving nominally unreactive NH_2_Cl as the chlorination source. Focus is placed upon the reactions of fluorescein, which undergoes facile ring chlorination by HOCl but reacts only very sluggishly with NH_2_Cl. This molecule was chosen because it has numerous advantages for mechanistic studies, including spectroscopic properties that make it amenable to detailed kinetic analyses, as well as easy identification of chlorinated reaction products. Imidazole-catalyzed ring chlorination of phenolic compounds is also demonstrated; this reaction could be portentous to interpretations of *in vivo* chlorination, since chlorotyrosines are now finding widespread use as stable markers of MPO-mediated oxidative stress in both intraphagosomal and extracellular environments [13,14,21].

## EXPERIMENTAL SECTION

### Materials

Chloramine (NH_2_Cl) solutions were prepared by reacting millimolar HOCl obtained from dilution of commercial bleach solutions with > 5-fold excess phosphate-buffered ammonia; ice-cold reactant solutions were flow-mixed using a 12-jet tangential mixer attached to a Harvard Apparatus 2000 syringe-drive unit. Hypochlorous acid and chloramine concentrations were routinely determined spectrophotometrically using ε_292_ = 350 M^−1^ cm^−1^ for OCl^−^ in strongly alkaline media [22] and ε_244_ = 429 M^−1^ cm^−1^ for NH_2_Cl [23]. The chloramine reagent concentrations were confirmed by analyzing their oxidizing capacity using Ellman’s reagent, in which 2-nitro-5-thiobenzoate (NTB) is oxidized to 5,5′-dithio(2-nitrobenzoate); ε_405_ = 1.36×10^4^ M^−1^ cm^−1^ was used to determine the amount of NTB consumed in the reaction [23]. These analyses indicated > 95% conversion of HOCl to NH_2_Cl. Imidazole (ImCl), N-α-acetylhistidine (NAHCl), 4-imidazoleacetic acid (IAACl) chloramines and the 1-methyl-3-chloroimidazolium cation (MeImCl^+^) were similarly prepared by reacting the parent amine with HOCl and their concentrations determined with Ellman’s reagent. However, since these chloramines (particularly MeImCl^+^) were relatively unstable and lost chlorinating capacity upon incubation at 37 °C, it proved convenient for kinetic studies with fluorescein at this temperature to prepare them immediately before reaction by using the 4-syringe mixing capability of the stopped-flow instrument. All other reagents were best-available materials obtained from commercial suppliers and were used as received. Reagent solutions were prepared from 18.1 MΩ water purified by passage through a Millipore Milli-Q Model Elix-S reverse osmosis/deionization unit.

### Methods

**Fig. 2:**
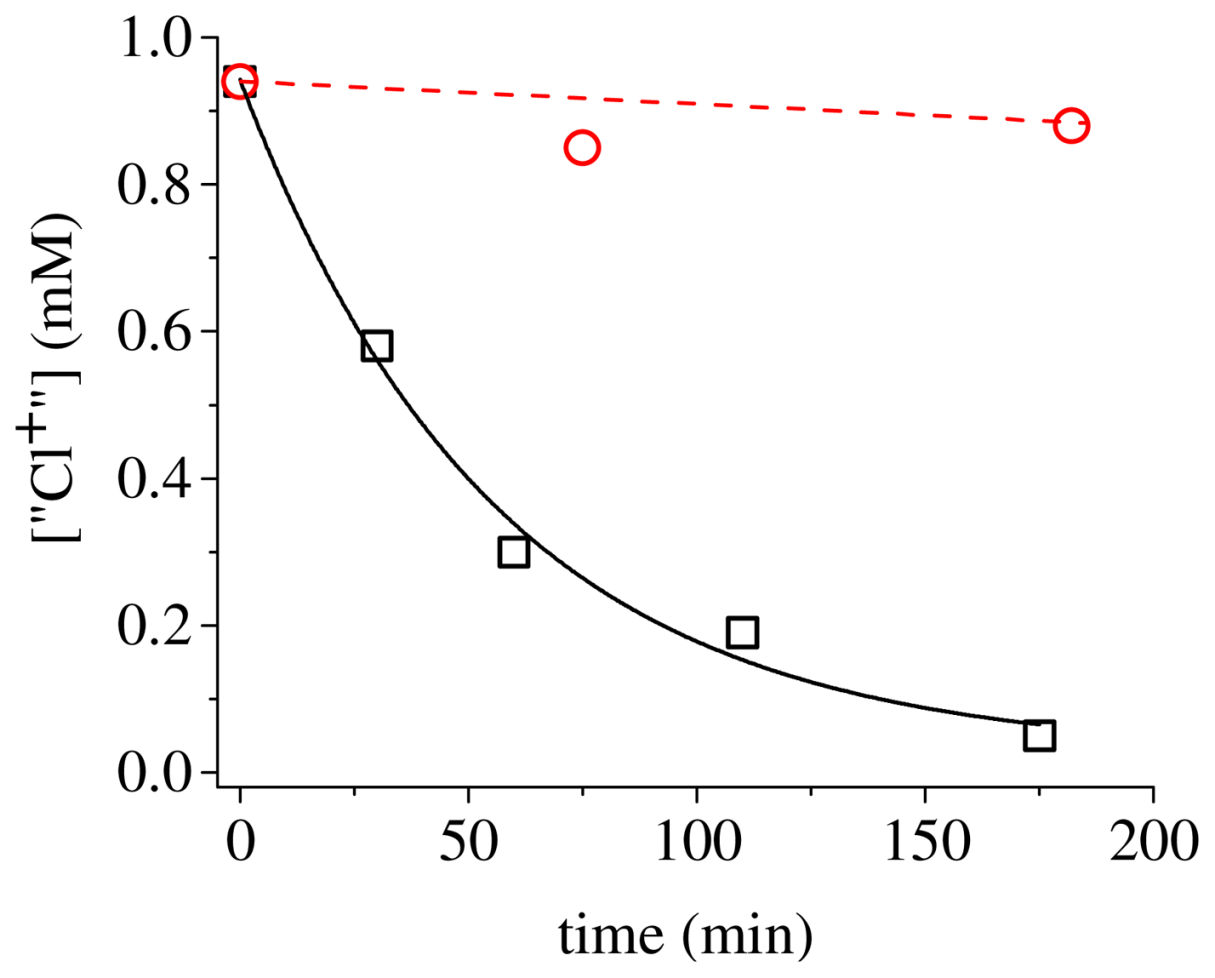
Decay of NH_2_Cl titer as measured by oxidation of NTB (Ellman’s reagent). Initial conditions: 0.94 mM NH_2_Cl in 50 mM P_i_ + 9mM NH_4_^+^, pH 7.4; reactions at 37 °C in the absence (circles) and presence (squares) of 20 mM NAH. The solid curve is a first-order fit to the NAH-catalyzed reaction; the dashed line is drawn considering that, when NAH was absent, ~33% of the NH_2_Cl had decayed after 18 h. The “Cl^+^” concentration measured at various times was identical to the NH_2_Cl concentration determined by direct spectrophotometric detection.

Optical spectra were obtained with a Shimadzu UV-2401PC instrument. Stopped-flow kinetics were obtained using an Applied Photophysics SX20 instrument with data collection/analysis using their ProData SX (vers 2.2.5.6)/ProData Viewer (vers 4.2.0) software. Reagent concentrations were adjusted so that first-order conditions were met in all quantitative studies; recorded kinetic traces were exponential to greater than four half-lives. (Typical curves are given in Figures 2, S2, S3, and S6.) The range of rate constants measured for repetitive runs on individual solutions was ≤ 5% for single-mixing experiments or ≤ 10% for multi-mixing experiments. Rate constants reported are the mean values of 5–10 runs whose error limits, expressed as average deviation from the mean, were ≤ 3%.

HPLC analyses were made using a Shimadzu LC10AD unit equipped with a SPD-M10A diode array detector and 250×4.6 mm 5 μm ultracarb ODS column; chromatograms were obtained by isocratic elution with 29% methanol/71% 20 mM P_i_, pH 7.4 at a flow rate of 0.3 mL/min. Mass spectrometric analysis of chlorofluoresceins was made using a LTQ-FT Ultra mass spectrometer (Thermo, San Jose, CA) in LTQ mode, with a Finnigan Ion Max API source set up for electrospray ionization in positive ion mode. Conditions: 5 kV spray voltage, 200 °C capillary temperature, 40 V capillary voltage, and 240 V tube lens voltage, 100–2000 m/z detection range. Analyte samples were adsorbed onto a C4 Ziptip, desalted with water, and eluted inline to the mass spectrometer at 20 μL/min with 50% ethanol, 50% water and 0.1% formic acid, as previously described [24]. Mass spectrometric analysis of chlorophenolic compounds was made using an ABSciex 4000 Q-trap LC/MS/MS system operated in the negative ion mode at 250 °C under the following conditions: CUR 30; tem 250; GS1 40; GS2 40; IS -4500; DP -45; EP -10. Sample was introduced into the mass spectrometer using a Shimadzu SIL-HTC liquid chromatograph equipped with a 3.5 μm Agilent Zorbax 300SB-C8 column (2.1×50 mm) operated at 0.2 mL/min total flow rate and 15 °C by applying a 5–90% gradient of H_2_O/CH_3_CN over 12 min; both eluants contained 0.1% formic acid.

## RESULTS

### Chloramine decomposition reactions

**Scheme 1:**
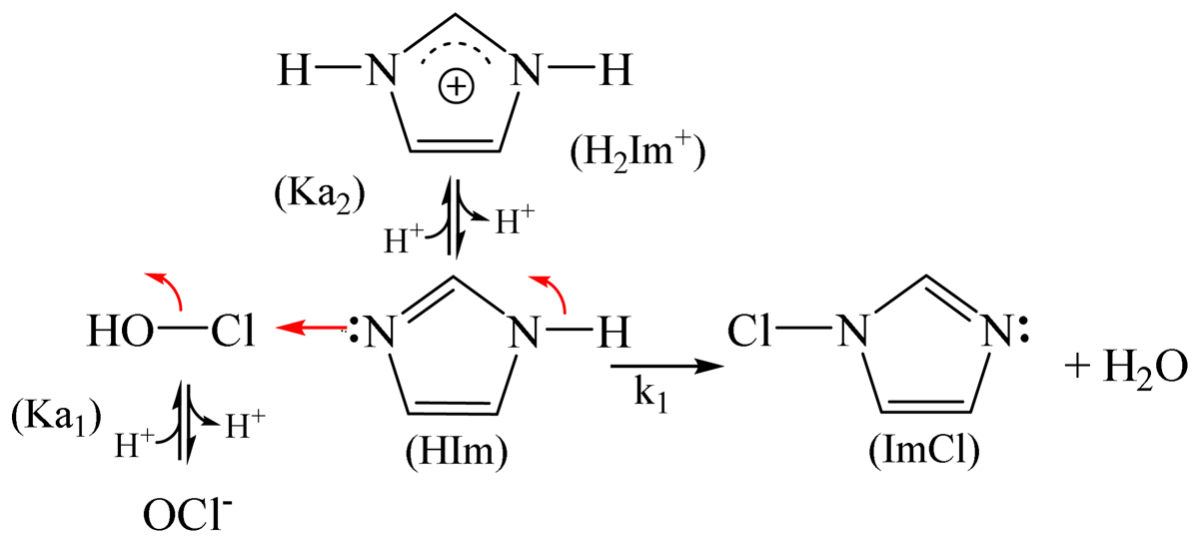
Pathway for imidazole chlorination by HOCl

When HOCl was mixed with 5-fold excess imidazole in phosphate buffers at pH 6–8, the OCl^−^ absorption band at 292 nm was immediately replaced by a broad featureless absorption tailing from higher energies that gave no maximum above 240 nm; absorption spectra at shorter wavelengths could not be obtained because imidazole gave very intense background absorption in this region. These spectral changes are similar to those previously reported for imidazole-containing compounds [4], and are consistent with formation of N-chloroimidazole (ImCl). Complete disappearance of the OCl^−^ band was observed, indicating that the reaction equilibrium lies far in favor of chloramine formation under these conditions. The ultraviolet “tail” slowly increased in intensity on a time scale that correlated with loss of chlorinating capacity (described below), consistent with ensuing degradation of ImCl. Rate constants for the initial reaction between HOCl and imidazole were determined by stopped-flow spectrophotometry by monitoring the loss in absorption intensity at 300 nm. With imidazole in (5–30)-fold excess, the reaction was simple first-order with apparent rate constants that were linearly dependent upon the imidazole concentration (Fig. S1), establishing the rate law: d(ImCl)/dt = k[Im]_T_[HOCl]_T_, where the subscripts T indicate total analyte concentrations, i.e., [Im]_T_ = [HIm] + [H_2_Im^+^], [HOCl]_T_ = [HOCl] + [OCl^−^]. The second-order rate constant is pH-dependent, exhibiting a weak maximum through the pH range 6–8 (Fig. S1). This profile is consistent with HOCl and the free base form of imidazole (HIm) being the true reactants, a result expected from the requirements for both a low-energy leaving group on the “Cl^+^” donor (OH^−^ vs. O^2−^) and an available lone pair on the acceptor [11,18,25] (i.e., Scheme 1). For this reaction, the pH-independent rate constant, k_1_ = k(1 + K_a1_/[H^+^])(1 +[H^+^]/K_a2_), where K_a1_ and K_a2_ are the acid dissociation constants for HOCl and H_2_Im^+^, respectively; assuming values of K_a1_ = 3.5×10^−8^ M (35 °C) [22] and K_a2_ = 1.0×10^−7^ M (20 °C), one calculates from a best-fit value to the data that k_1_ ≈ 1.7 (± 0.3)×10^5^ M^−1^ s^−1^ (37 °C in 100 mM phosphate, I = 0.5 M (Na_2_SO_4_)). (A list of the reactions investigated in this study is given in Table S1 along with the numbered rate constants that identify them and pertinent rate data.) The rate law predicts that, at fixed reactant concentrations, the rate of ImCl formation is optimal at pH 7.2. A similar pH dependence has been reported for the rate of reaction of HOCl with 4-imidazole acetic acid (IAA); the maximal rate constant reported here was k_IAA_ = 1.2×10^5^ M^−1^ s^−1^, measured at 22 °C in 100 mM phosphate [4]. Rate constants reported for other imidazole-containing compounds are also very similar [20].

**Scheme 2:**
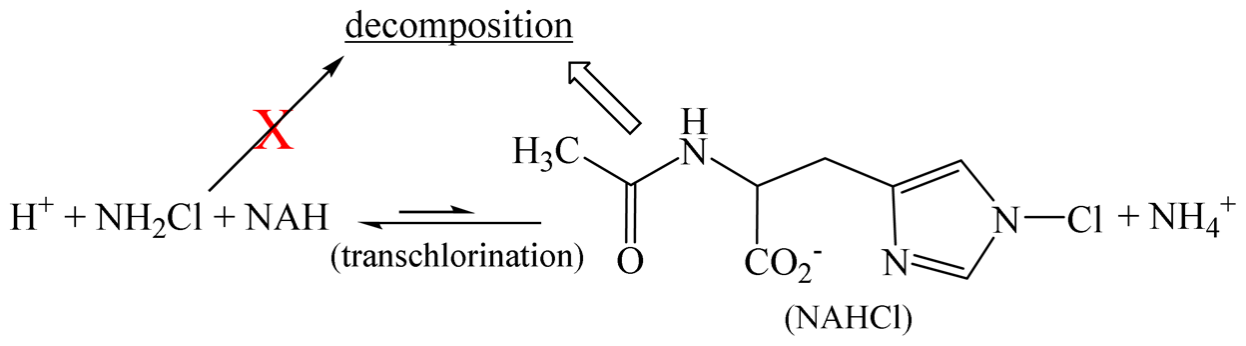
Imidazole-catalyzed pathway for chloramine decomposition

Imidazole chloramine underwent decomposition fairly rapidly, as indicated by loss of chlorinating capacity toward fluorescein (described below) and loss of oxidizing titer with NTB. As measured with NTB, decay of “Cl^+^” was exponential, with t_1/2_ values that ranged from 43–75 min in various media at pH 7.4 and 37 °C. Pretreating buffers and reactant solutions by passage through Chelex columns and rinsing glassware in nitric acid did not alter the decay rates, suggesting that participation of adventitious metal ions in these reactions was negligible. Decay of the chloramine formed from the histidine model compound, imidazoleacetic acid (IAA), was also briefly examined. In Chelex-treated 50 mM P_i_, pH 7.4, loss of “Cl^+^” from solutions containing IAACl occurred at 37 °C with an apparent t_1/2_ ~ 60 min; under these conditions, the decay was not simple first-order, however. In contrast to these imidazole-based chloramines, NH_2_Cl is quite stable, undergoing only very slow loss in oxidizing titer with an apparent t_1/2_ ≥ 18 h under comparable reaction conditions (Fig. 2). However, this decay was markedly accelerated when N-acetylhistidine (NAH) was included in the reaction medium; this reagent was chosen because earlier reports had indicated that its imidazole chloramine is relatively unstable [19,20,26,27]. In the presence of 20 mM NAH, the decay of NH_2_Cl was accelerated by at least 25-fold (Fig. 2), based upon the relative reaction half-times (~40 min vs. ~18 h). A plausible explanation for this phenomenon is that the relatively unstable NAH chloramine forms to a small extent, opening a second, more reactive channel for chloramine decay, e.g., as illustrated in Scheme 2:

The total chlorinating capacity ([“Cl^+^’]), as determined by reaction with NTB, was identical within experimental uncertainty to [NH_2_Cl] determined by direct spectrophotometric measurement. Assuming a transchlorination mechanism of this type, this correspondence indicates that either the transchlorination equilibrium position lies far to the left, precluding direct detection of NAHCl, or the spectra of the two chloramines are indistinguishable in the accessible spectral region. The latter explanation is less likely because similar chloramine transfer reactions between aminoacyl compounds [28] and between IAACl and alkyl amines or aminoacyl compounds [19] have demonstrated sufficient changes in spectral properties that the transchlorination equilibria and kinetics could be determined.

Yet more dramatic effects were observed when NAH was replaced by 1-methylimidazole (MeIm). Chlorination of this compound is expected to give the 1-methyl-3-chloroimidazolium cation (MeImCl^+^), which is a structural analog of the imidazole chloramine protonated at the remote nitrogen atom, i.e.:

(1)
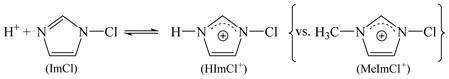


When pH 7.4 phosphate-buffered solutions containing millimolar HOCl were flow-mixed with 10-fold excess MeIm, there was a rapid loss of oxidant titer, as measured by NTB analysis. At 37 °C, this reaction was complete within the minimal time required to carry out the analysis (~1 min), and ~80 % of the oxidant had been consumed by this time even when ice-cold solutions were reacted. The product spectra gave only a broadly increasing absorption with increasing energy in the ultraviolet region that was reminiscent of the absorption spectra of imidazole chloramine and its degradation products. The intensity of this ultraviolet absorption decreased slightly over the ensuing 30 min, suggesting slow continuing degradative reactions were at play. The rate of loss of oxidizing capacity was determined by stopped-flow spectrophotometry by monitoring the OCl^−^ absorption band at 300 nm. These reactions were carried out at 37 °C in a reaction buffer comprising 100 mM phosphate, pH 7.0, I = 0.5 M (Na_2_SO_4_), with [HOCl] = 0.6–1.25 mM and [ImCl] in (10–60)-fold excess. The decay profile was exponential, indicating first-order dependence upon HOCl, with t_1/2_ values ranging from 0.1–10 s under the prevailing conditions. The experimentally determined rate constants exhibited simple second-order dependence upon [MeIm] (Fig. S2), indicating a rate law of the form, dP/dt = k[HOCl]_T_[MeIm]_T_^2^, where k = 1.4 (±0.2)×10^3^ M^−2^ s^−1^. Because a termolecular reaction is highly improbable, the rate law implies that the decay is a multistep process involving formation of at least one reaction intermediate. A likely mechanism involves formation of MeImCl^+^, followed by its oxidative degradation of a second MeIm in a bimolecular reaction, i.e.:

(2)
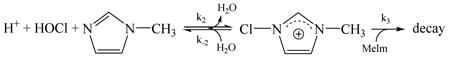


Under conditions where the HOCl-MeImCl^+^ equilibrium is rapidly established, k_3_ ≪ k_2_, k_-2_ and the reaction follows the observed rate law with k = K_2_k_3_, where K_2_ = k_2_/k_-2_ is the HOCl-MeImCl^+^ transchlorination equilibrium constant and k_3_ is the second-order rate constant for the reaction of MeImCl^+^ with MeIm. Wavelength-dependent stopped flow studies over the range λ = 260–310 nm gave no evidence for accumulation of a distinct intermediate, as would be indicated by transient absorption phenomena. Consequently, as with the NH_2_Cl-NAH reaction, the equilibrium position for the HOCl-MeImCl^+^ transchlorination step must lie far to the left, that is, at equilibrium, [HOCl] ≫ [MeImCl^+^]. Since K_2_ is unknown, k_3_ cannot be independently determined from the data. These data also do not give any information on the chlorinating capacity of the MeImCl^+^ reaction intermediate. However, in a subsequent section we will describe multimixing experiments that demonstrate that it is even more reactive than HOCl as a chlorinating agent toward fluorescein.

### Fluorescein chlorination reactions

**Fig. 3:**
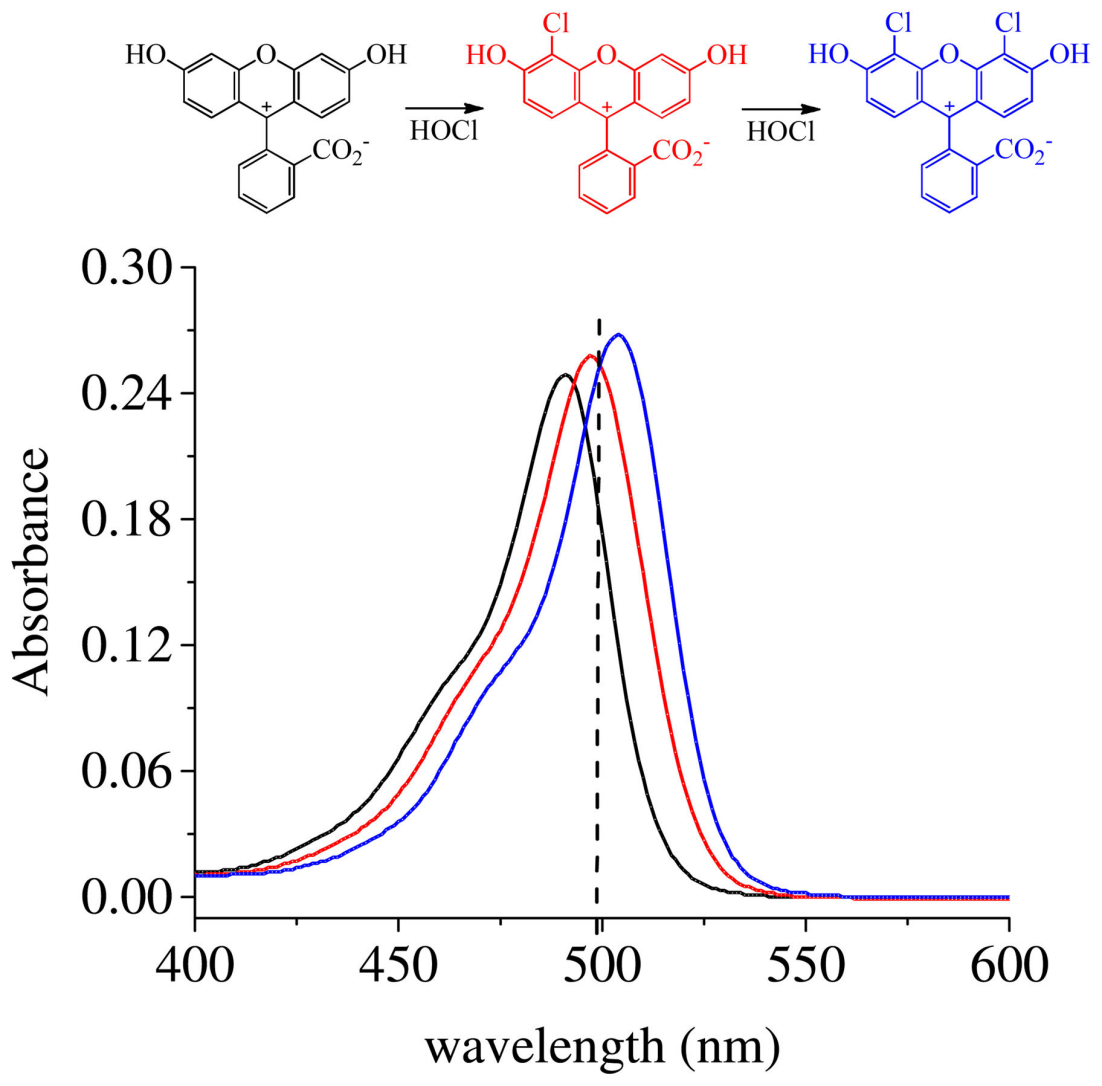
Visible absorption spectra of fluorescein (black) and its 4′-monochloro- (red) and 4′,5′-dichloro (blue) derivatives [29] prepared by flow-mixing fluorescein with 1.0 equivalent and 2.0 equivalents HOCl, respectively. Conditions: 0.5 mM fluorescein in 25 mM phosphate, pH 7.4. The dashed vertical line at 500 nm shows the pH–dependent isosbestic point for flCl and flCl_2_, denoted λ_isos_ in the text. Molecular structures are shown over the spectra.

When exposed to HOCl, fluorescein (fl) undergoes sequential ring chlorination to form 3′-monochloro (flCl) and 3′5′-dichloro derivatives (flCl_2_), which are characterized by small progressive bathochromic shifts in their intense visible absorption and fluorescence bands (Fig. 3); further reaction of flCl_2_ with HOCl leads to destruction of the chromophore. Because chlorination of the ring deactivates it toward electrophilic attack, the rate constants for these reactions decrease in the order: fl → flCl → flCl_2_ → chromophore bleaching. Consequently, titrimetric addition of HOCl yields first flCl, then flCl_2_ as the major reaction products.

Previous work had shown that the reactions between HOCl and fluoresceins are moderately rapid, although only the rate constant for the flCl → flCl_2_ step was determined in these studies [29]. When [HOCl] > [fl], spectrally determined kinetic waveforms for the HOCl/fluorescein reaction are highly wavelength dependent (Fig. S3).^2^ A useful condition for examining the first step, HOCl + fl → flCl, is the isosbestic wavelength (λ_isos_) for flCl and flCl_2_. Here, the spectrophotometer is blind to the second step, flCl → flCl_2_; since the first step is temporally well resolved from the bleaching reactions (see below), it is possible to observe at λ_isos_ the first step essentially uncoupled from all subsequent reaction steps. Because flCl has protic equilibria in the weakly acidic domain [30], λ_isos_ is acid-dependent below pH 8. Consequently, it was determined experimentally for all reaction conditions by overlaying spectra containing varying ratios of the two compounds at constant total fluorescein. (For pH values of 6.0, 6.5, 7.0, and ≥ 8.0, λ_isos_ were at 491, 497, 499, and 500 nm, respectively; individual component spectra followed Beer’s law.) The possibility that equilibration of the chlorinated fluoresceins, i.e., fl + flCl_2_ ↔ 2flCl, might contribute to the reaction dynamics was also explored by comparing the spectral stabilities of solutions containing flCl and equimolar fl plus flCl_2_. No changes in these spectra were observed for periods up to 3 h, even when the media (pH 7.0, 250 mM P_i_) contained 25 mM imidazole, confirming that transchlorination equilibration between the fluoresceins did not contribute to the observed reaction dynamics.

At λ_isos_, flCl formation caused an increase in absorbance; with HOCl in large excess, the reaction gave an exponential growth profile for at least 5 half-lives under all reaction conditions examined (Fig. S3). The pseudo-first order rate constants characterizing these curves were linearly dependent upon [HOCl], yielding the rate law: d[flCl]/dt = k_4_[fl]_T_[HOCl]_T_; in 100–210 mM phosphate, pH 7.0, I= 0.5 M (Na_2_SO_4_), k_4_ = 4–5×10^3^ M^−1^ s^−1^ at 37 °C. Reaction of flCl prepared by flow-mixing stoichiometric amounts of fl and HOCl was independently studied; here it was convenient to use 512 nm, the absorption maximum for flCl_2_, as monitoring wavelength. Analysis gave a rate law of the same order, i.e., d[flCl_2_]/dt = k_5_[flCl]_T_[HOCl]_T_, where k_4_/k_5_ = 2.2 in identical reaction media (Fig. S4). Bleaching of flCl_2_ by excess HOCl did not follow either simple first- or second-order rate laws, but the kinetic traces could be fitted to a biexponential equation; unlike the chlorination reactions, the pseudo-first order rate constants obtained exhibited a second-order dependence upon [HOCl]. From this rate behavior it is evident that flCl_2_ bleaching is a complex, multistep process. Reaction half-times for bleaching were >30-fold larger than those for fluorescein chlorination under all conditions used for these studies; since they were noninterfering, these reactions were not studied further.

**Fig. 4:**
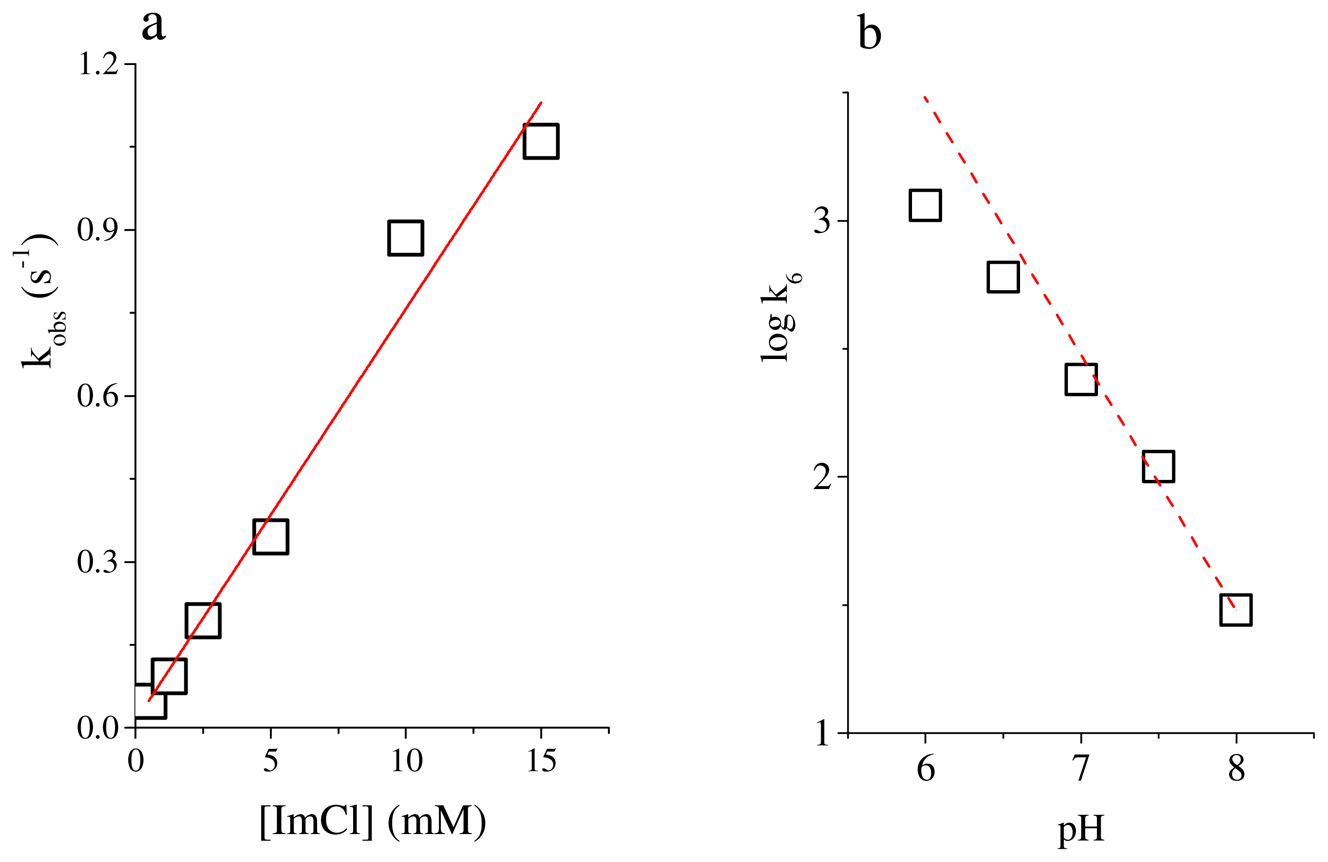
Kinetic summary for the reaction between ImCl and fluorescein to form flCl. Panel a: pseudo-first order rate constant obtained by reacting 5 μM fluorescein with variable amounts of ImCl in 25 mM imidazole/25 mM phosphate at pH 7.4, 37 °C; λ_isos_ = 500 nm. The ImCl was formed in the ageing loop of a 4-syringe mixer by premixing HOCl with the imidazole-containing buffer. The red line is a linear least-squares fit to the data, for which k_6_ = 75 M^−1^ s^−1^ (where k_obs_ = k_6_[ImCl]). Panel b: pH dependence of k_6_ obtained at I = 0.5 M, 37 °C. The dashed red line gives the theoretical expectation for simple first order dependence upon [H^+^].

The reaction between ImCl and fluorescein was studied by utilizing the 4-syringe mixing capability of the stopped-flow instrument. This procedure was adopted to obviate incubation of ImCl reactant solutions at 37 °C for extended periods and thereby risk degradation of the chloramine. The imidazole chloramine was generated *in situ* in the ageing loop by mixing HOCl with an excess of Im, following which the product solution was reacted with fluorescein. Reaction with ImCl exhibited the same progression of spectroscopic changes in fluorescein as that generated by HOCl, albeit at a slower rate of change. Under pseudo-first order conditions with the chlorinating agent in excess, the rapid growth in absorption corresponding to flCl formation closely followed first-order kinetics at λ_isos_ to beyond 5 half-lives, where the experimentally-determined rate constant (k_obs_) is the sum of the pseudo-first order rate constants for concurrent reactions by all of the chlorinating species present in solution, i.e., k_obs_ = Σk_RCl_[RCl]_T_. The only chlorinating agents (RCl) expected for these reactions are HOCl and ImCl (with possibly some ImCl_2_ at very low [Im]_T_/[HOCl]_T_ ratios [23]). Ageing times were chosen to ensure that the reactions between HOCl and Im (described above) reached equilibrium before mixing with fluorescein. This condition was verified by demonstrating that rates of fluorescein chlorination were independent of time delay intervals over a wide range (typically 0.5–20s). When [Im]_T_/[HOCl]_T_ = 10–100, k_obs_ was also independent of [Im]_T_. This indicates that the equilibrium position for the reaction given in Scheme 1, i.e., for which K_Im_ = [ImCl]_T_/[HOCl]_T_[Im]_T_, lies sufficiently far toward ImCl formation that reaction with HOCl contributes negligibly to the overall reaction, i.e., [ImCl]_T_ ≫ [HOCl]_T_ and k_obs_ = k_4_[HOCl] + k_6_[ImCl] ≈ k_6_[ImCl]. Under these conditions, k_obs_ was linearly dependent upon [ImCl]_T_ (Fig. S5), allowing determination of k_6_. These second-order rate constants increased with acidity over the measured range (pH 6–8), with a near-linear dependence upon [H^+^] (Fig. 4). Because an earlier report had indicated that reaction between NH_2_Cl and nitrite ion exhibited general acid catalysis in phosphate buffers [17], we carefully examined the phosphate concentration dependence of measured rate constants using Na_2_SO_4_ as an inert electrolyte where necessary to maintain constant ionic strength. No systematic [P_i_] dependence was detected except in the very weakly buffering solutions at pH 8.0; this latter effect was traced to small increases in pH during the overall reaction. It therefore appears that the pH dependence reflects solely specific acid catalysis, i.e., a reaction pathway in which protonation precedes rate-limiting fluorescein chlorination. Although protonation sites exist on both ImCl and fluorescein [30], reactivity of the latter is not expected to be strongly influenced by its protonation state. In contrast, protonation of the remote N on ImCl should greatly weaken the N-Cl bond and strongly facilitate “Cl^+^” transfer. Specific acid catalysis therefore implies that HImCl^+^ is the actual chlorinating species, a notion that is pursued further in the following section. Because the pH dependencies of the reactions of HOCl and ImCl with fluorescein differ, their rate constant ratios when compared under a common set of conditions are pH-dependent. In the present studies, where direct comparisons were made, k_4_/k_6_ varied from ~20 to ~300 (Fig. S5). These ratios are consistent with previous studies in which the relative chlorinating activities of HOCl and imidazole chloramines were determined [19].

When [Im]_T_/[HOCl]_T_ ≤ 10, k_6_ was no longer independent of [Im]_T_, but increased as the [Im]_T_/[HOCl]_T_ ratio decreased. This effect was studied most carefully at pH 7, where a 2.6-fold rate constant increase was achieved when [Im]_T_/[HOCl]_T_ reached 1.1. A reasonable explanation for this increase is that conversion of HOCl to ImCl is not complete under these conditions, i.e., since k_obs_ = k_4_[HOCl]_T_ + k_6_[ImCl]_T_, equilibrated solutions now contain a sufficient amount of HOCl to influence the rate of fluorescein chlorination. Here, the previously determined values of k_4_ and k_6_ can be used to determine [HOCl]_T_, [ImCl]_T_ and [Im]_T_ from their stoichiometric relationships and the measured values of k_obs_ under the prevailing conditions, thereby allowing an estimate of K_Im_. Analysis of the data (summarized in Supplementary Information) gives K_Im_ = 8.9 (±1.4)×10^4^ M^−1^ at 37 °C in 212 mM phosphate, pH 7.0, (I = 0.50 M). Assuming that HOCl and HIm are the only reactant protonation states that lead to ImCl formation (Scheme 1) and that ImCl is the dominant form of the imidazole chloramine at neutral pH, the corresponding pH-independent equilibrium constant, K_Im_’ = [ImCl]/[HOCl][HIm] is given by the relationship: K_Im_’ = K_Im_(1 + K_a1_/[H^+^])(1 + [H^+^]/K_a2_), for which K_Im_’ ≈ 3×10^5^ M^−1^.

**Fig. 5:**
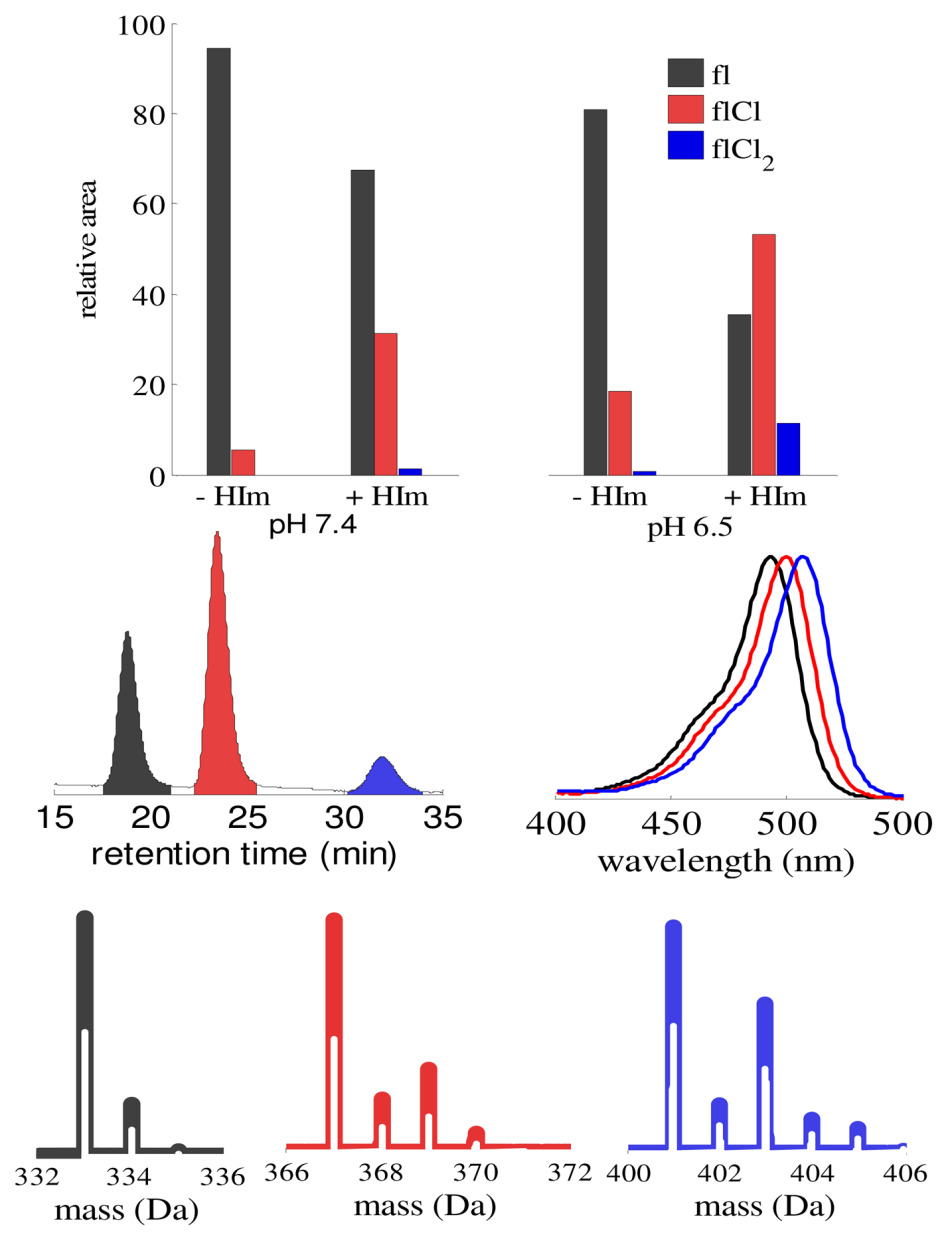
Product identification from imidazole-catalyzed reactions of NH_2_Cl with fluorescein. Reaction conditions: at pH 7.4, 5 μM fluorescein and 180 μM NH_2_C in 50 mM phosphate incubated at 37 °C for ~1 h in the presence of 40 mM imidazole or ~2 h when imidazole was absent; at pH 6.5, same reaction conditions, except imidazole was 10 mM when present and an incubation period of ~2 h was used for both solutions. The top panels show distributions of fl, flCl and flCl_2_ as determined by the relative areas of the HPLC peaks. The center left panel shows a representative chromatogram for the pH 6.5 product solution (imidazole present) obtained at a detection wavelength of 500 nm and the center right panel shows the normalized optical absorption spectra for each of the three chromatographic peaks determined using a diode array detector. Apart from a small red shift, these correspond to authentic spectra of fl, flCl and flCl_2_ (cf. fig. 3). The lower panels display LTQ-FT mass spectra of the three HPLC isolates; experimental masses are shown in black with theoretical spectra for fl, flCl, and flCl_2_, respectively, overlaid at half height in white. Mass error of all isotope peaks shown is < 1 ppm; peaks were widened for clarity.

Simple chloramines, including NH_2_Cl [6], are much less reactive toward fluorescein, yielding negligible chlorinated products on timescales where reaction with HOCl is complete. However, when imidazole was present in the reaction medium at millimolar concentration levels, spectral shifts in the absorption maxima consistent with formation of flCl and flCl_2_ were observed. These changes occurred over a period of 1–2 h at 37 °C and were accompanied by loss of NH_2_Cl, as measured by NTB oxidation. HPLC chromatograms revealed peaks whose retention times and visible absorption spectra were identical to authentic flCl and flCl_2_; structures were confirmed by FT-ICR mass spectrometry, which gave ion clusters at m/z 367 and 401amu exhibiting the expected isotope distribution patterns for the monochloro- and dichloro derivatives, respectively (Fig. 5). The extent of chlorination achieved was greater at pH 6.5 than 7.4, and increased with increasing [Im]_T_, consistent with a mechanism involving *in situ* formation of ImCl as the chlorinating agent. Small amounts of flCl could also be detected in acidic media at higher NH_2_Cl concentrations when imidazole was omitted, but the fluorescein chlorination was clearly imidazole-catalyzed under these conditions. Unlike reactions in Im-containing solutions, there was no appreciable degradative loss of NH_2_Cl over the incubation period when Im was absent.

### The special case of the 1-methyl-3-chloroimidazolium (MeImCl)^+^cation

One approach to identifying the protonation site in an acid-catalyzed reaction is to substitute a nondissociable methyl group at the presumed basic site, a method known as “fixing the proton” [31]. If reactivity comparable to the underivatized compound is observed, the methylated site is also presumed to be the site of protonation. In the present case, the relevant methylated analog is 1-methylimidazole (reaction 1). Studying its reactivity is complicated, however, by the rapid loss of chlorinating capacity in solutions containing HOCl and MeIm, a condition which necessitated use of stopped-flow spectrophotometry with multi-mixing capabilities.

**Fig. 6:**
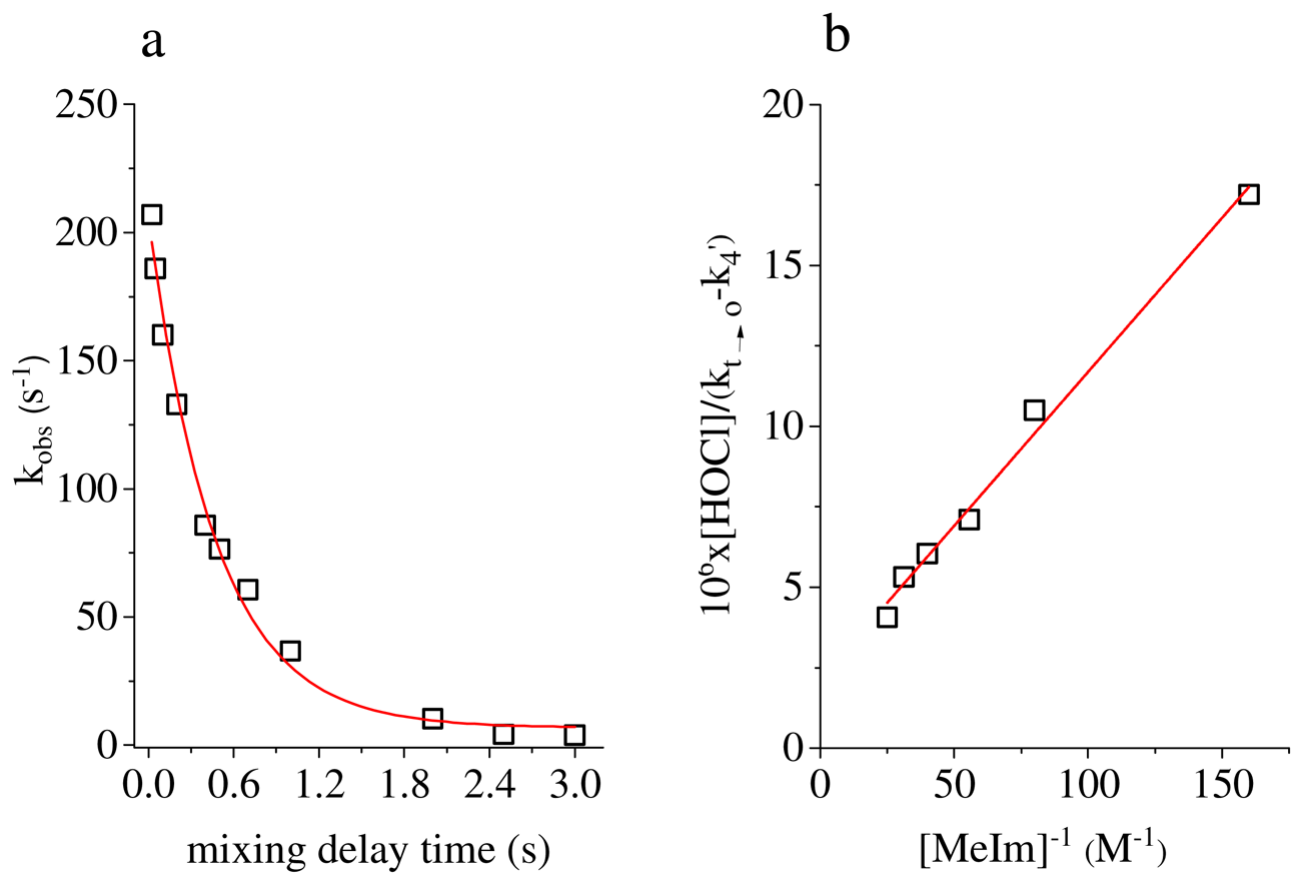
Chlorination of fluorescein by MeImCl^+^. The cationic chloramine was prepared in a 4-syringe apparatus by reaction of HOCl with MeIm, following which the product solution was mixed with fluorescein. Panel a: dependence of pseudo-first order rate constant for flCl formation upon the delay time interval between MeImCl^+^ formation and its reaction with fluorescein. Conditions: 10 μM fluorescein, 25 mM MeIm, 1.25 mM HOCl (initial) in 210 mM phosphate, pH 7.0, 37 °C (I = 0.5 M), λ_isos_ = 499 nm. The red curve is an exponential fit to the data, for which k = 0.44 s^−1^. Physically, this represents the loss of chlorinating capacity during incubation of the HOCl-MeIm reactant solution. Panel b: linearized form of rate data for reaction of MeImCl^+^ with fluorescein according to the mechanism given in equation 3 (see text for derivation); k_t→0_ is the experimentally determined rate constant extrapolated to a mixing time delay of 0 s and k_4_’ = 4.6 s^−1^ is the independently determined pseudo-first order rate constant for direct reaction of HOCl with fluorescein. The red line is the linear least-squares fit to the data.

Solutions undergoing rapid MeIm-catalyzed decay of HOCl were capable of chlorinating fluorescein to yield flCl and flCl_2_ products, as identified by the diagnostic bathochromic shifts in fluorescein visible absorption spectra and mass spectrometric identification of the molecular ions; this chlorinating capacity was lost upon incubation for a period sufficient to completely degrade the HOCl. Stopped-flow spectrophotometry made with fluorescein as the limiting reagent indicated that the fl → flCl step (measured at λ_isos_) was strictly first-order in fluorescein (Fig. S6). Because the decay of chlorinating capacity under these conditions was rapid, the apparent first-order rate constant (k_obs_ = Σk_RCl_[RCl]_T_, where now RCl are HOCl and MeImCl^+^) was highly dependent upon the preincubation time for the reaction between HOCl and MeIm in the ageing loop. Specifically, under a given set of reaction conditions, the measured first-order rate constant (k_obs_) decreased exponentially with increasing incubation time at a rate corresponding to loss in chlorinating capacity of the solution (Fig. 6a), that is, with the same kinetics as described in the section entitled “chloramine decomposition reactions” (Fig. S2). One conclusion from the decomposition studies is that the HOCl-MeImCl^+^ transchlorination equilibrium lies far toward HOCl under these conditions. Consequently, the “chlorinating capacity” is very nearly equal to the hypochlorous acid concentration. Thus, the exponential decrease in k_obs_ with increasing delay time shown in Fig. 6a parallels the exponential loss of HOCl in the ageing loop prior to mixing with fluorescein, indicating that the rate constant for fluorescein chlorination is also first-order in [HOCl].

By extrapolating the values of k_obs_ measured at various incubation times, one could determine its value at t = 0, the time immediately after mixing (and for which the reactant concentrations were well known). The extrapolated initial rate constants (k_t→0_) increased with increasing amounts of MeIm in the reactant solutions (where [MeIm]/[HOCl] = 5–30). Under these conditions, k_t→0_ was 15–70 times greater than the fluorescein chlorination rate constant of HOCl alone (measured in identical solutions from which MeIm was omitted). These results clearly implicate in the chlorination reaction a MeIm-based intermediate that is more reactive than HOCl itself. The data were quantitatively analyzed according to the mechanism shown in equation (3), which is an expansion of equation (2) that now includes in addition to HOCl decay the concurrent chlorination of fluorescein by direct reaction with HOCl (the k_4_ pathway) and by reaction with the imidazole chloramine intermediate (the k_7_ pathway). Application of the steady-state

(3)
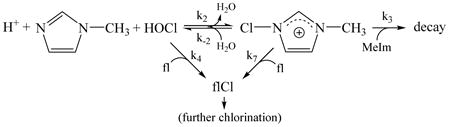


approximation to MeImCl^+^ yields the following complete rate equation:

$$d[\text{flCl}]/\text{dt}=\{k_{4}+k_{2}k_{7}[\text{MeIM}]/(k_{-2}+k_{3}[\text{MeIM}]+k_{7}[\text{fl}])\}[\text{HOCl}][\text{fl}].$$

This equation correctly predicts the first-order dependence for flCl formation upon [HOCl], as demonstrated by the ageing time-dependence studies (Fig. 6a); the observation that the rate is first-order in fluorescein requires that k_7_[fl] ≪ k_-2_ + k_3_[MeIm]. With this approximation, the initial rate constant is k_t→0_ = {k_4_ + k_2_k_7_[MeIm]/(k_-2_ + k_3_[MeIm])}[HOCl]). At the relatively high [MeIm] used in some of the experiments, the k_2_[MeIm] denominator term cannot be ignored, and the data can be linearized only by plotting the function in reciprocal form; specifically, [HOCl]/(k_t→0_ – k_4_′) = k_3_/k_2_k_7_ + k_-2_/k_2_k_7_[MeIm]^−1^ (Fig. 6b). Note that k_4_’ is the pseudo-first order rate constant for direct reaction of HOCl with MeIm, i.e., k_4_’ = k_4_[HOCl]. These data do not allow determination of the equilibrium constant for MeImCl^+^ formation (k_2_/k_-2_); however, the intercept/slope ratio of the plot in Fig. 6b (23 M^−1^) is equal to k_3_/k_-2_. Thus, k_3_[MeIm]/k_-2_ ≤ 0.2 at [MeIm] ≤ 10 mM, validating the pre-equilibrium approximation applied to the analysis of the kinetics of MeIm-accelerated decay of HOCl. As before, since the transchlorination equilibrium constant (k_2_/k_-2_) is unknown, k_7_ cannot be uniquely determined from the data. However, the relative rate constants for reaction of MeImCl^+^with fluorescein and MeIm can be estimated from the kinetic data; ratioing k_2_k_7_/k_-2_ and k_2_k_3_/k_-2_ (Table S1) gives k_7_/k_3_ ≈ 7×10^3^.

### Chlorination of phenolic rings

**Fig. 7:**
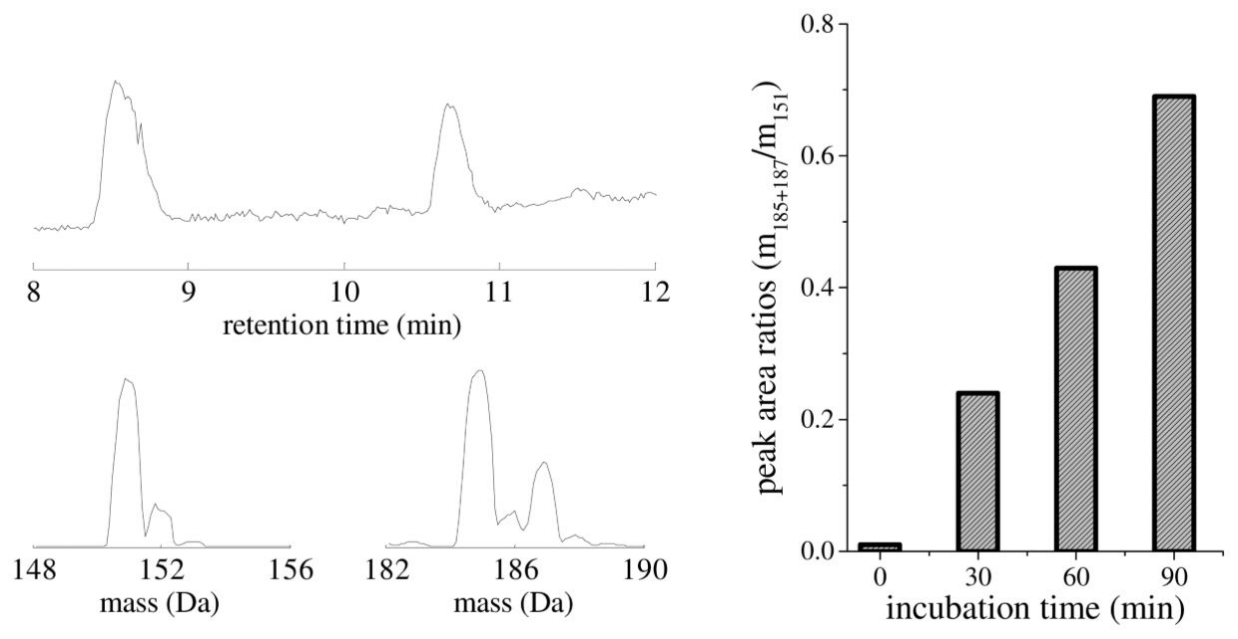
HPLC/MS/MS analysis of product solutions from imidazole-catalyzed reaction of NH_2_Cl with p-hydroxyphenylacetic acid (HPA). Left panels: 0.5 mM NH_2_Cl and 0.5 mM HPA incubated in 20 mM imidazole, 25 mM phosphate, pH 7.0, for 90 min at 37 °C, following which 0.5 mM dithiothreitol was added to quench the reaction. The chromatogram based upon total ion current (TIC) revealed two major species with retention times of ~ 8.5 and ~10.7 min under the prevailing elution conditions (upper trace). The mass spectrum of the more rapidly eluting peak (lower left) was dominated by the molecular ion of unreacted starting material (HPA - H^+^, m/z = 151 Da); the dominant feature of the other peak was an ion whose mass (185 Da) and isotopic distribution (185/187 = 3/1) corresponded to a monochlorinated product (lower right). When imidazole was omitted, the chromatograms of analogous incubated solutions exhibited only a single peak at ~ 8.5 min, whose mass corresponded to that of unreacted HPA (151 Da). Right panel: relative integrated ion currents for chromatographic peaks at m/z (185 + 187) vs m/z 151 at the indicated incubation times.

The capacity of imidazole to catalyze reaction of phenolic compounds with chloramine was briefly examined. Specifically, 0.5 mM 4-hydroxyphenylacetic acid (HPA) was reacted with an equal amount of NH_2_Cl in 20 mM imidazole or 50 mM phosphate, pH 7.0, for timed intervals, then quenched with ~ 20% excess dithiothreitol and product solutions were analyzed by HPLC/mass spectrometry. In imidazole buffer, a progressive increase in the monochlorinated product (m/z = 185/187 (3/1)) was observed relative to the unreacted HPA (m/z = 151), so that by 90 min incubation about one-half of the HPA had been chlorinated (Fig. 7). In contrast, in phosphate buffer, there was no detectable chlorination by NH_2_Cl over the same time period, consistent with earlier reports [12,20]. Formation of more extensively chlorinated products was not observed in either medium.

## DISCUSSION

### The chemical basis for catalysis

Hypochlorous acid, chloramines and other chlorinating agents vary widely in their reactivities toward reaction partners. These reactions are generally considered to occur by pathways involving direct transfer of the chlorinium cation (“Cl^+^”) to an acceptor site, effecting net exchange for H^+^ or two-electron oxidation of the reaction partner. Accordingly, important rate-determining factors are: (1) the availability of suitable orbitals (e.g., lone pairs) on the chlorine acceptor molecule to form a nascent bond to facilitate “Cl^+^” transfer, (2) the relative electrophilic and nucleophilic character of chlorine donor and acceptor molecules, respectively, and (3) the identity of the donor leaving group. Specific reactions that have been proposed to be initiated by “Cl^+^” transfer include oxidation of inorganic anions (I^−^, Br^−^, CN^−^, NO_2_^−^, SO_3_^2−^) [32], hydrogen peroxide [25], and sulfhydryl compounds [33] by HOCl, as well as oxidation of inorganic anions (I^−^, SO_3_^2−^, NO_2_^−^) by NH_2_Cl [17], oxidation of H_2_O_2_ by various chloramines [34], and amine transchlorination reactions [35]. Also, the magnitudes of rate constants for chlorination of simple amines by HOCl have been shown to parallel the amine basicities [18], as expected for a “Cl^+^”-transfer mechanism.

An early attempt to quantify the chlorinating capacity of these compounds focused upon the relative strengths of the N-Cl bond as revealed in their equilibrium constants for hydrolysis to HOCl, e.g., R_2_NCl + H_2_O → R_2_NH + HOCl. Here the “chlorine potential” for R_2_NCl was defined as the negative logarithm (or pK_CP_) of the equilibrium constant. Typical values for ranged from 5–14, where lower numbers indicate higher chlorine potentials [36]. Based upon our analysis of the kinetics of reaction between HOCl-generated ImCl fluorescein, we estimate from K_Im_’ (= K_PC_^−1^) that pK_CP_ for ImCl is ~5.5, making it one of the most reactive of the chloramines for which this sort of analysis is available. The equilibrium constant for the transchlorination reaction, HIm + NH_2_Cl → ImCl + NH_3_, can also be calculated from K_Im_’ and the corresponding constant estimated for reaction of ammonia with HOCl,^1^ from which one obtain K_trans_ = K_Im_’/K_f_ = [ImCl][NH_3_]/[HIm][NH_2_Cl] ≈ 3×10^−6^. As with other chloramine reactions, the equilibrium distribution of “Cl^+^” is highly dependent upon the medium acidity. Assuming that the NH_3_ and HIm free bases are the only reactive species, the apparent equilibrium constant (K_trans’_ = [ImCl]_T_[NH_3_]_T_/[HIm]_T_[NH_2_Cl]_T_) is given by K_trans_(1 + [H^+^]/K_a3_)/(1+ [H^+^]/K_a2_), where K_a3_ = 6.3×10^−10^ M is the acid dissociation constant for NH_4_^+^. Accordingly, the [ImCl]/[NH_2_Cl] ratio will increase nearly linearly with acidity through the range pH 10–7, then become pH-independent below pH ~6. At pH 7, K_trans_’ ≈ 10^−2^, so that equilibrium solutions containing extensively chlorinated ammonia will also contain imidazole chlorinated to the extent of a few percent of the total imidazole pool. Thermodynamic analysis indicates that the equilibrium position between ImCl and HOCl reflected in K_Im_’ is determined almost entirely by differences in their N-Cl and O-Cl bond strengths (Fig. S7), whereas the equilibrium position between ImCl (or HOCl) and NH_2_Cl also reflects the greater N-H bond strength of NH_3_ relative to HIm (and to the O-H bond strength in HOCl). Specifically, the transchlorination equilibrium would be displaced far toward NH_2_Cl formation if it were not for the much greater proton affinity of the amide anion (NH_2_^−^) relative to the imidazolate anion (Im^−^). This difference is in large part attributable to the capacity of Im^−^ to delocalize charge over its molecular framework. Similarly, the increase in ImCl/NH_2_Cl ratio predicted to occur over the pH 11–6 range is driven solely by the greater basicity of NH_3_ over HIm.

Although the greater reactivity of ImCl over NH_2_Cl can be attributed in part to the much weaker N-Cl bond in the former chloramine, the thermodynamic analysis does not consider that the chlorination reactions of both these compounds are acid-catalyzed. The origins of this kinetic factor can be understood by considering the transchlorination reactions of NH_2_Cl. Here, the rate constant-pH profiles for the amine transchlorinations are bell-shaped, presenting a formal ambiguity with respect to the site of protonation within the transition-state complex [35]. Specifically, the predominant forms of the reactants at most accessible pH values are NH_2_Cl and the protonated amine, RNH_3_^+^. These are unlikely to be the reactant pair, however, because RNH_3_^+^ possesses no available lone-pair orbitals to accept “Cl^+^” and transfer from NH_2_Cl would require release of NH_2_^−^, a very strongly basic (pK_a_ ≈ 34 [37]), hence, poor leaving group. The alternative possibility, that NH_3_Cl^+^ and RNH_2_ are the actual reactants, preserves the form of the rate law and overcomes these energetic restrictions, i.e., now RNH_2_ is a good nucleophile with an available lone-pair donor orbital and NH_3_ is the leaving group associated with “Cl^+^” transfer from NH_3_Cl^+^. Utilizing the known acid dissociation constants for NH_3_Cl^+^ and RNH_3_^+^, the Margerum group has calculated that the bimolecular rate constant for Cl^+^ transfer from NH_3_Cl^+^ can be as high as 2×10^8^ M^−1^ s^−1^, i.e., approaching the diffusion-controlled limit [17]. The actual observed rate constants are ~10^8^-fold lower than this, however, because the concentration of the reactant pair ([NH_3_Cl^+^][RNH_2_]) is vanishingly small under all solution conditions, which in turn is a consequence of the large difference in protonation constants for NH_2_Cl and RNH_2_. Consistent with this interpretation, Prütz has reported that trimethylamine, when present in catalytic amounts, markedly accelerates HOCl reactions with a phenolic compound (salicylate), a compound containing olefin bonds (sorbate) and nucleotide heterocyclic bases [38]. Since (CH_3_)_3_NCl^+^ contains no dissociable protons whose loss will deactivate the complex (i.e., the protons are “fixed”), one infers from its apparent high rate of reaction as a chlorinating agent that NH_3_Cl^+^ will also be highly reactive. Analogous chemistry had also been established in earlier studies for reaction of HOCl with hydrogen peroxide [25,31]. Here, although the predominant forms of reactants in solution are H_2_O_2_ and OCl^−^, the actual reactants are HO_2_^−^ and HOCl; the lower kinetic barrier for reaction between the latter pair arises because OH^−^ is a much better leaving group than O^2−^, hence the proton in the transition state complex is bound to OCl^−^ rather than HO_2_^−^.

The reaction between ImCl and fluorescein also exhibits a near-linear dependence upon acidity (Fig. 4), indicating the requirement for a proton in the reaction transition state. Ambiguity concerning the site of protonation arises in this case because the fluorescein dianion is also basic and has protic equilibria in weakly acidic environments [30]. However, by analogy with the “Cl^+^” transfer reactions of HOCl and NH_2_Cl, it is reasonable to suggest that ImCl is activated by protonation. In this case, the N atom in the imidazole ring remote from the N-Cl bond presents an alternative binding site that can still increase polarization within the N-Cl bond through a combination of inductive and resonance effects (equation 1). This site may be considerably more basic than the one containing the N-Cl bond and protonation here, in addition to increasing the electropositive character of the Cl atom, would allow release of HIm upon “Cl^+^” transfer, rather than Im^−^, a much poorer leaving group. We were unable to determine the protonation constant for ImCl in these studies. To test the notion that HImCl^+^ is a reactive chlorinating agent, we “fixed” the proton with an electronically equivalent but nondissociable methyl group. As expected, the MeImCl^+^ cation generated by reaction of 1-methylimidazole with HOCl was much more reactive toward fluorescein chlorination than HOCl itself, validating these general reactivity principles. *To summarize, the much greater chlorinating capacity of ImCl over NH_2_Cl as revealed in their reactions with weak nucleophiles can be ascribed to the much weaker intrinsic N-Cl bond in ImCl and the relative ease of forming a highly reactive chlorammonium cation (HImCl^+^) by protonation at an alternate basic site (*equation 1*). The difference in N-Cl bond energies between ImCl and NH_2_Cl is nearly compensated by the difference in proton affinities of the conjugate bases of the amines which drives the “Cl^+”^ transchlorination equilibrium in neutral solutions toward ImCl formation (*Fig. S7).

### Imidazole chloramine formation and reactions in biological environments

The rate law for reaction between HOCl and imidazole, including both rate constant and pH dependence, is nearly identical to that previously determined for histidine model compounds [4]. These rate constants, which are comparable to those for reactions of cysteine disulfide and many primary amino groups in neutral media [18], fall in the intermediary range for HOCl reactions with biological compounds, where only sulfhydryl and thioether groups [4,5,39,40] and highly nucleophilic chromophoric centers [41] are more reactive. From estimates of the protein concentration in plasma [4] and neutrophil phagosomes [5] (~2 mM), the concentration of protein histidyl groups in these environments is estimated to be 5–15 mM [20,42]. The total primary amino group concentration from all sources has been estimated at ~50 mM in serum and ~150 mM in the neutrophil cytosol [23]. Thus, after depletion of more reactive nucleophilic sites, the higher concentrations of primary amines should dictate that continued reactions of HOCl will lead primarily to formation of simple chloramines. Modeling studies using plasma levels of free amino acids have suggested that a few percent of the histidyl groups will be directly chlorinated by HOCl [20] within a pool of relatively unreactive primary chloramines; this chloramine pool should then serve to maintain via transchlorination equilibration the more reactive imidazole chloramines as they are consumed by available nucleophiles (Fig. 1). Based upon the NH_2_Cl-ImCl transchlorination equilibrium constant (K_trans_’) and estimated concentrations of the various biological amines*,* conversion of 20% of the primary amines to chloramines would be expected to generate at equilibrium 10–40 μM imidazole-based chloramines. This level of amine chlorination appears achievable within the neutrophil phagosome following activation, where nearly all O_2_ consumed by stimulated respiration generates HOCl [5,6]. However, the extent of chloramine formation in extracellular environments, where the amount of myeloperoxidase-generated HOCl may be considerably less, is more difficult to gauge.

Because K_trans_’ is insensitive to medium acidity below pH 7, comparable levels of imidazole chloramines should be formed throughout the physiological range. Their selectivity toward biological targets could vary widely, however. In these studies, the rate of fluorescein chlorination was acid-dependent over the range pH 6–8 (Fig. 4) and the much greater rate of MeImCl^+^ decay over ImCl implies a strong acid dependence for ImCl decay as well. Comparison of the reaction rate constants for imidazole and fluorescein with MeImCl^+^ (equation 3), which is almost 10^4^-fold greater for fluorescein than imidazole (Table S1), suggests that the intrinsic reactivity of HImCl^+^ will also be highly dependent upon physiological reaction partners. More subtle factors, such as imidazole chloramine mobilities and proximity effects could also influence reactivity patterns; for example, because MeImCl^+^ decay is imidazole-dependent, rates of decay of protein-bound, hence relatively isolated, histidyl chloramines might be significantly retarded. Addressing these issues will require kinetic investigation of a broader range of reaction partners.

Imidazole catalysis of chlorination has been demonstrated experimentally in the present study, where we have shown that histidine model compounds promote chloramine decay via a mechanism that involves decomposition of the histidine chloramine itself and that phenolic groups, including fluorescein and a tyrosyl model compound, are chlorinated by NH_2_Cl in imidazole-catalyzed reactions. These data extend earlier work by Pattison and Davies on relative rates of biological oxidations by HOCl and histidine chloramine model compounds and on chlorination of N-acetytyrosine by various types of biological chloramines [19], from which it was concluded that histidyl protein groups are uniquely reactive and can constitute important secondary oxidants in HOCl-induced protein damage. These reactions might also contribute to the slow chlorination of protein tyrosyl groups attending bacterial phagocytosis by neutrophils described by Chapman et al., which continued well beyond 60 min post-phagocytosis, i.e., long after cessation of MPO-catalyzed HOCl formation [13]. One should note, however, that involvement of protein side-chain histidyl groups in transchlorination reactions has not yet been unambiguously demonstrated. Alternatively, studies with model peptides have shown that side chain lysine chloramines can mediate tyrosine chlorination when the secondary structure allows close approach of these groups [43] and tyrosine chlorination has also been observed in a tetrapeptide devoid of lysine and histidine (Gly-Gly-Tyr-Arg) [12].

### Biological implications

The biological significance of these reactions remains to be determined. Long ago, Thomas and coworkers demonstrated that NH_2_Cl was as equally effective as HOCl at killing *Escherichia coli* in suspension cultures, and that cellular death correlated with oxidation of accessible sulfhydryl groups, implying that the lethal lesions were generated by reaction of the chlorinating agents with easily oxidizable target molecules [8–10]. Moreover, bacterial killing by neutrophils is often rapid, virtually coinciding with phagocytosis and the respiratory “burst” [7]. Rate constants for HOCl oxidation of thiols are in the 10^7^–10^8^ M^−1^ s^−1^ range [40], approaching the encounter-controlled limit for aqueous solutions. Rate constants for reactions with biological chloramines are on the order of 10^2^–10^3^ M^−1^ s^−1^ [26], which is 10^4^–10^5^ fold lower, but still sufficiently large to oxidize millimolar concentrations of sulfhydryl compounds within a few seconds exposure time. Reactions of chlorinium ions are also inherently rapid [17,36,38]. However, for chloramines such as NH_2_Cl and ImCl that exist primarily in their neutral conjugate base forms at physiological pH levels, apparent rate constants for reaction of the corresponding chlorinium ions will not be large because they are the product of an unfavorable protic equilibrium constant and a large rate constant for reaction, e.g., k_obs_ = K_a_×k_HImCl+_. Following depletion of HOCl and the most reactive target molecules, the remaining chlorinating activity within the reaction zone, e.g., the neutrophil phagosome, will be low [5]. Not until these conditions are reached will the catalytic potential of imidazole chloramines become evident, e. g., as has been illustrated in the present case with phenolic ring chlorinations.

As discussed above, there is presently no obvious role for imidazole catalysis in MPO-mediated bactericidal action within the neutrophil phagosome. However, the yeast *Saccharomyces cerevisiae* is more refractory and, unlike bacteria, can be rescued if antioxidants are added within several minutes following exposure to lethal doses of HOCl [44]. Although the fungicidal mechanisms have not been identified, it is possible that maintenance of a greater chlorinating potential in the form of imidazole chloramines facilitates cell death in these instances. More subtle effects can be anticipated in extracellular media, where chloramines can act as potent regulators of cell metabolism and access to intracellular environments may depend upon transchlorination reactions among members of the chloramine pool [26, 28]. Where elevated levels of imidazole chloramines can be maintained, they could influence cellular response by altering product distributions. For example, Winterbourn and associates have shown that HOCl can oxidize glutathione (GSH) irreversibly to sulfonamides and thiosulfonate, [45] whereas reaction with simple chloramines is limited to formation of the disulfide (GSSG) and (in cells) mixed protein-glutathione disulfides [26], which are products from which GSH can be regenerated. In more complex environments, catalysis by more reactive imidazole chloramines should diminish these differences.

Efficient catalysis of chlorination by primary amines in biological settings may not be limited to imidazole-containing compounds. Notably, Prütz has shown that TMP and UMP chlorinated at ring nitrogen positions oxidize biological substrates at rates that approached those of HOCl, whereas the chlorinating capacity of the CMP analog (presumably chlorinated at its primary amino substituent) reacted much more sluggishly [46]. Consequently, thymidine- and uridine-containing biomolecules could potentially function in similar catalytic cycles. As previously noted, trimethylamine reacts wth HOCl to form an extremely reactive chlorinium ion [38]; however, (CH_3_)_3_N is a dietary derived chemical that apparently does not accumulate in the plasma of normal humans above low micromolar levels [47].

### Conclusion

Imidazole can catalyze “Cl^+^” transfer reactions of primary chloramines with relatively poor nucleophiles through intermediary formation of imidazole chloramines. The chemical basis for the enhanced reactivity of the latter is a relatively weak N-Cl bond and its capacity to undergo protonation at the second basic site on the imidazole ring to form the highly reactive chloramine cation, HImCl^+^. This protonation not only further weakens the N-Cl bond, but also lowers the activation barrier for “Cl^+^” transfer by allowing the catalyst to be directly regenerated in its lowest energy protic state, i.e., HIm. Despite the weaker N-Cl bond in ImCl vs. NH_2_Cl, the much greater proton affinity of the amide anion (NH_2_^−^) over imidazolate (Im^−^) drives the transchlorination equilibrium toward ImCl formation, so that solutions of NH_2_Cl (and other primary chloramines) in physiological environments can be expected to contain a few percent of imidazole chloramines. Upon protonation to the corresponding chloramine cations, these become extremely reactive chlorinating agents.

## Supplementary Material

supplement

## Highlights

- Imidazole catalyzes chlorination of phenols by NH_2_Cl and chloramine decomposition.
- The active catalyst is the conjugate acid of imidazole chloramine (HImCl^+^).
- Biological chlorination reactions generate catalytically relevant amounts of ImCl.
- The chemical basis for catalysis by the imidazole group is discussed.

## Footnotes

1 (The equilibrium constant for chloramine formation, defined as K_f_ = [NH_2_Cl]/[NH_3_][HOCl] for the reaction: HOCl + NH_3_ → NH_2_Cl + H_2_O, is estimated to be K_f_ ~ 10^11^ M^−1^ [17]. At pH 7, assuming an effective total amine concentration of 1–10 mM, the equilibrium ratio of chloramine to HOCl within the phagosome would be on the order of [NH_2_Cl]/[HOCl] = 10^5^–10^6^. This ratio is also dependent upon protic equilibria among the reactants; specifically, because only the acidic form of HOCl and the free base forms of the amines are reactive [18], the [NH_2_Cl]/[HOCl] ratio will depend upon the solution pH and identities of endogenous amines. Acid dissociation constants do not vary greatly for amines with alkyl and aminoacyl substituents, however, so that their equilibrium ratios are expected to be similar to that determined for ammonia and HOCl.)

2 (For example, at wavelengths where flCl absorption is dominant, one observes an initial rise in absorbance, corresponding to its formation from fl; this is followed by absorbance decreases, corresponding to formation of flCl_2_ and the subsequent chromophore bleaching; conversely, where fl absorption dominates, one observes only multiphasic loss of absorption as the reaction proceeds through the sequence of steps.)
