# Supplementary material for: Imidazole catalyzes chlorination by unreactive primary chloramines
Source: Free Radic Biol Med. Author manuscript; Available in PMC 2016 May 1. (PMC4387080; doi:10.1016/j.freeradbiomed.2015.01.026)
Supplement: supplement [file NIHMS661165-supplement.docx]

*Supplementary Data*:

“Imidazole catalyzes chlorination by unreative primary chloramines”

M.D. Roemeling, J. Williams, J. S. Beckman, and J. K. Hurst

Table S1. Numerical assignments of chemical reactions and rate data:^a^

(1) HOCl + HIm → ImCl + H_2_O; k_1_ = 1.7×10^5^ M^-^1 s^-1^

(37^o^ C, 0.1M P_i_, I = 0.5 M)

(2) HOCl + MeIm → MeImCl^+^ + OH^-^; k_2_ (see reactions 3,7)

(-2) MeImCl^+^ + OH^-^ → HOCl + MeIm; k_-2_ (see reactions 3,7)

(3) MeImCl^+^ + MeIm → decomposition k_3_ k = k_2_k_3_/k_-2_ = 1.4×10^3^ M^-2^ s^-1^

(37^o^ C, 0.1M P_i_, pH 7.0, I = 0.5 M)

(4) HOCl + fl → flCl + H_2_O k_4_ = 4-5×10^3^ M^-1^ s^-1^

(37^o^ C, 0.1-0.2 M P_i_, pH 7.0, I = 0.5 M)

(5) HOCl + flCl → flCl_2_ + H_2_O k_5_ = 1.9×10^3^ M^-1^ s^-1^

(37^o^ C, 0.1 M P_i_, pH 7.0, I = 0.5 M)

(6) ImCl + fl → flCl + HIm k_6_ = 2.4×10^2^ M^-1^ s^-1^

(37^o^ C, pH 7.0, I = 0.5 M—reaction is pH and ionic strength dependent—cf. fig. 4)

(7) MeImCl^+^ + fl → flCl + MeIm + H^+^ k_7_ k_2_k_7_/k_-2_ = 1.0×10^7^ M^-2^ s^-1^

(37^o^ C, in pH 7.0 P_i_, I = 0.5 M)

____________________________________________________________________________

^a^Rate constants are accurate to better than ±10%.

Kinetic determination of K_Im_ for the transchlorination equilibrium:

HOCl + HIm = H_2_O + ImCl

K_Im_ = [ImCl]/[HOCl]_T_[HIm]_T_,

where the subscript T refers to total analyte concentration under the prevailing conditions (pH 7.0, I = 0.5 M, 37 ^o^C), i.e., [HOCl]_T_ = [HOCl] + [OCl^-^] and [HIm]_T_ = [HIm] + [H_2_Im^+^].

k_obs_ = k_HOCl_[HOCl]_T_ + k_ImCl_[ImCl],

where k_HOCl_ = 5.4×10^3^ M^-1^ s^-1^ and k_ImCl_ = 3.0×10^2^ M^-1^ s^-1^

Let the total initial added HOCl be defined as Cl_T_, where Cl_T_ = [HOCl]_T_ + [ImCl];

Similarly, define Im_T_ = [HIm]_T_ + [ImCl]. Then, k_obs_ = k_HOCl_(Cl_T_ -[ImCl]) + k_ImCl_[ImCl];

rearranging gives [ImCl] = (k_HOCl_Cl_T_ – k_obs_)/(k_HOCl_ – k_ImCl_), from which [HOCl]_T_ and [HIm]_T_ can be determined.

| k_obs_ (s^-1^) | Im_T_ (mM) | [ImCl] (mM) | [HOCl]_T_ (mM) | [HIm]_T_ (mM) | K_Im_ (mM^-1^) |
| --- | --- | --- | --- | --- | --- |
| Cl_T_ = 0.50 mM |  |  |  |  |  |
| 0.186 | 1.56 | 0.493 | 0.007 | 1.067 | 66 |
| 0.214 | 1.10 | 0.487 | 0.013 | 0.613 | 60 |
| 0.253 | 0.825 | 0.480 | 0.020 | 0.345 | 70 |
| 0.289 | 0.675 | 0.473 | 0.027 | 0.202 | 87 |
| 0.379 | 0.55 | 0.455 | 0.045 | 0.095 | 106 |
| Cl_T_ = 0.25 mM |  |  |  |  |  |
| 0..091 | 1.0 | 0.247 | 0.003 | 0.753 | 109 |
| 0.121 | 0.50 | 0.241 | 0.009 | 0.259 | 103 |
| 0.149 | 0.40 | 0.235 | 0.015 | 0.165 | 95 |
| 0.186 | 0.325 | 0.228 | 0.022 | 0.097 | 107 |
|  |  |  |  |  | K_avg_ = 89 (±14) |

Fig. S1. Dynamics of reaction between HOCl and imidazole. Left panel: Pseudo-first order rate constants for OCl^-^ disappearance (k_obs_) with imidazole in excess, monitored at 300 nm; conditions: 100 mM P_i_ at the indicated pH values, μ_i_ = 0.50 M (Na_2_SO_4_), 37 ^o^C. Right panel: second-order rate constants (k_Im_) determined at various pH values (shaded circles) compared to a theoretical curve made assuming that HOCl and ImH are the reactive protonation states.

Fig. S2. Reaction of HOCl with 1-methylimidazole. Conditions: 100 mM P_i_, pH 7.0; μ_i_ = 0.5 M (Na_2_SO_~~4~~_); 37 ^o^C. Panel a: representative kinetic trace obtained with 1.25 mM HOCl and 12.5 mM MeIm; the red curve is the exponential data fit with k_1_ = 0.226 s^-1^. Panel b: linear dependence of k_1_ upon the square of the methylimidazole concentration; the red line is a least-squares fit to the data, for which k_3rd_ = 1.4(±0.2)×10^3^ M^-2^ s^-1^.

Fig. S3. Wavelength dependence of kinetic waveforms for the reaction between HOCl and fluorescein. Left panel: detection at the flCl absorption maximum (λ = 496 nm); right panel: detection at the flCl/flCl_2_ isosbestic point (λ = 500 nm). The dashed vertical lines in the insets show these wavelengths in relation to the visible absorption bands of fluorescein and the chlorinated products. Conditions: 100-210 mM P_i_, pH 7.0 (μ_i_ = 0.5 M (NaSO_4_), 37 ^o^C.

Fig. S4. Reactions of fluoresceins with HOCl. Pseudo-first order rate constants (k_obs_) plotted against the concentration of reactant in excess. Conditions: 37^o^ C, 0.1 M P_i_, pH 7.0, I = 0.5 M. *Rate constants determined from the slopes of the plots are: k_4_ = 4.3×10^3^ M^-1^ s^-1^ (black); k_5_ = 1.9×10^3^ M^-1^ s^-1^ (red); k_flCl2_ = 2.2×10^4^ M^-2^ s^-1^ (blue). The rate constant given for flCl_2_ bleaching is the faster of the two rate constants determined from a biphasic fit to the data.*

Fig. S5. Comparative reactivities of HOCl and ImCl with fluorescein. Pseudo-first order rate constants (k_obs_) plotted against excess chlorinating agent concentration under identical medium conditions, i.e., I(P_i_) = 0.5 M, pH 7.0, 37 ^o^C. Second-order rate constants obtained from the slopes were k_4_ = 5.4×10^3^ M^-1^ s^-1^ and k_6_ = 2.9×10^2^ M^-1^ s^-1^ (k_4_/k_6_ = 19).

Fig. S6. Reaction between the1-methylimidazole chloramine cation and fluorescein. 1-MeImCl^+^ was formed by reaction between HOCl and 1-methylimidazole. Following an 0.5 s delay to allow equilibrium between the chlorinating agents to be established, the product solution was mixed with fluorescein and its conversion to 3’-monochlorofluorescein was monitored at 299 nm. Initial reaction conditions (after complete mixing): 25 mM MeIm, 1.25 mM HOCl and 10 μM fluorescein in pH 7.0 P_i_ buffer (μ_i_ = 0. 5 M); 37 ^o^C. Under these conditions, t_1/2_ for decay of the chlorinating capacity of the solution is ~1 s. The red line is an exponential data fit with k_1_ = 71 s^-1^.

Fig. S7: Thermodynamic cycles for transchlorination equilibria involving ImCl. ΔG^o^ is the equilibrium free energy, given by ΔG^o^ = ΔG_1_ + ΔG_2_ – ΔG_3_, where ΔG_1_ is the free energy for proton dissociation from HIm, ΔG_3_ is the corresponding constant for the reaction partner, and ΔG_2_ is the difference in bond energies for ImCl and the reaction partner. Values for ΔG_1_ and ΔG_3_ were calculated from reported values for the acid dissociation constants for HIm (pK_a1_ = 14.4), H_2_O (pK_3_ = 15.7), and NH_3_ (pK_a3_ ≈ 34) [37] using the relationship, ΔG = 2.3RTpK_a_. ΔG^o^ was similarly calculated from the overall equilibrium constants estimated in this work. Values obtained from cycle A are ΔG^o^ = -10.3kcal/mol and ΔG_2_ = -8.4 kcal/mol, indicating that the driving force for ImCl formation is very nearly accounted for by the difference in R-Cl bond energies. In contrast, values obtained from cycle B (ΔG^o^ = 5.8 kcal/mol and ΔG_2_ = 33 kcal/mol) indicate that the N-Cl bond in ImCl is much weaker than in NH_2_Cl, but is nearly completely compensated for by the very high proton affinity of the amide anion (NH_2_^-^).
